# Effects of a new magnetostrictive ultrasonic scaler and a traditional piezoelectric ultrasonic scaler on root surfaces and patient complaints

**DOI:** 10.1038/s41598-024-57037-7

**Published:** 2024-03-19

**Authors:** Xiaoqing You, Xiaohong Wu, Shiwei Chen

**Affiliations:** Fujian Biological Materials Engineering and Technology Center of Stomatology, School and Hospital of Stomatology, Fujian Medical University, 246 Yangqiao Zhong Rd, Fuzhou, 350002 Fujian China

**Keywords:** Periodontal disease, Scaling, Piezoelectric ceramics, Magnetostriction, Ultrasonic therapy, Ultrasonic treatment, Oral hygiene, Medical research, Clinical trial design

## Abstract

Tooth wear and pain are the primary concerns of patients undergoing periodontal scaling. The aims of this study were to compare the effects of a new magnetostrictive ultrasonic scaler and a traditional piezoelectric ultrasonic scaler on tooth surface roughness and calculus removal and to determine their impacts on patient discomfort during supragingival cleaning. This article had two parts: an in vitro study and a clinical study. In the in vitro study, thirty teeth with subgingival calculus were randomly assigned to two scaling treatment groups: magnetostrictive scalers (n = 15) and piezoelectric scalers (n = 15). Surface roughness measurements were taken at baseline and after scaling, and the root samples were visualised by SEM after scaling. Additionally, a single-centre randomised split-mouth clinical trial was conducted. Eighty-five participants diagnosed with chronic gingivitis or periodontitis were randomly assigned to receive supragingival scaling. The magnetostrictive scaler was used in half of the mouths (n = 85), and the piezoelectric scaler was used in the other half of the mouths (n = 85). Data on pain, noise, and vibration were collected using a VAS questionnaire, and the operating time was recorded. In both in vitro and clinical studies, magnetostrictive scalers were reported to be more effective than piezoelectric scalers in removing dental deposits (P < 0.05). Additionally, the root surface after scaling with the magnetostrictive scaler was smoother than that after scaling with the piezoelectric scaler in the in vitro study (P = 0.02). SEM examination also revealed that fewer dental materials were lost after instrumentation with the magnetostrictive scaler than after instrumentation with the piezoelectric scaler. Piezoelectric scalers caused less discomfort to patients in terms of pain, noise, and vibration than magnetostrictive scalers (P < 0.05). According to this clinical study, the magnetostrictive scaler caused more discomfort during supragingival scaling than the piezoelectric scaler. Moreover, the magnetostrictive scaler was also more efficient and produced a smoother root surface with less material loss after scaling than the piezoelectric scaler, as demonstrated in the in vitro study.

## Introduction

Dental plaque biofilms cause inflammation and damage to periodontal tissues^1^. The removal of plaque biofilms and calculus from the tooth surface is necessary for successful periodontal treatment^2^. While auxiliary therapies are available for treating periodontal disease, such as ozone, photobiomodulation, antibacterial chemical, and probiotic treatments^3–5^, traditional scaling and root planing (SRP) remains the gold standard^6,7^. Traditional SRP can be achieved using either manual or powered scalers^8–10^. Ultrasonic scalers use high-frequency mechanical vibration, hole effects and microacoustic flow to remove bacterial plaque and calculus and promote periodontal tissue health^2^. Clinicians prefer ultrasonic scalers to other scalers due to their high efficiency^9,11,12^. Compared to manual instruments, ultrasonic instruments are less invasive and preserve more cement^13^. However, ultrasonic periodontal therapy can cause patient discomfort, including toothache and psychological distress^8,14^. Some patients refuse necessary treatment due to fear of pain and discomfort during the procedure^15^. The noise and vibration generated by ultrasonic devices can also increase anxiety and fear^16^. Therefore, dentists should choose minimally invasive and comfortable treatments for patients^4^.

Several researchers have examined the impacts of various periodontal scalers on root surfaces, including their morphological and biological properties^9,11,17–20^. However, the findings remain inconclusive. Busslinger et al. and Muhammed et al. reported that a piezoelectric scaler produces a rougher surface than a magnetostrictive ultrasonic scaler^9,15^. Yousefimanesh et al. reported that using a piezoelectric scaler with 200 g of lateral force results in smoother surfaces than using a magnetostrictive scaler with the same lateral force^18^. The morphology of the root surface not only affects the preservation of healthy cementum but also impacts biofilm aggregation^21,22^, periodontal cell attachment and periodontal tissue healing^23^. The goal of periodontal treatment is to preserve the surface material and morphology of the affected tooth while removing plaque biofilms in a minimally invasive manner. Currently, chemical agents, such as probiotics, ozone, and oxygen, that can help maintain oral health are available, but their use is always related to mechanical plaque removal^5,24^.

Previous reports have demonstrated that different periodontal devices result in varying levels of comfort due to their different operating principles^25–27^. There are two categories of ultrasonic scalers: magnetostrictive and piezoelectric. Magnetostrictive scalers generate tip vibrations from 18 to 45 kHz, while piezoelectric scalers generate tip vibrations from 25 to 50 kHz^27^. Muhney et al.^25^ reported that piezoelectric ultrasonic scalers are more effective at removing calculus and cause less discomfort during the scaling process than magnetostrictive ultrasonic scalers. In contrast, Japanese researchers^26^ observed that patients treated with magnetostrictive ultrasonic scalers experience less discomfort than those treated with piezoelectric ultrasonic scalers. However, scholars have yet to reach a definitive conclusion on which type of ultrasonic scaler is most comfortable for patients.

The fourth oral epidemiological survey in China revealed that more than 90% of adults suffer from periodontitis and calculus. Calculus is prevalent in the population and poses a threat to oral health^28^. Manufacturers are constantly updating cleaning equipment to improve and effectively remove calculus. A new magnetostrictive ultrasonic scaler (Bangvo Technology Co., Ltd., China) with a titanium alloy working tip has recently been used for scaling and root planning. However, the effects on periodontally affected tooth surfaces and patient comfort after scaling with the Bangvo scaler have not yet been investigated. The objective of this study is to compare the effects of a magnetostrictive scaler and piezoelectric scaler (EMS Piezon Master 700, Switzerland) on tooth surfaces and patient complaints during supragingival scaling. The hypothesis is that supragingival cleaning with magnetostrictive instruments will result in root surface morphologies and patient discomfort levels similar to those resulting from cleaning with piezoelectric instruments. In this study, the ultrasonic scaler that can best maintain tooth integrity and improve patient comfort is identified.

## Methods

The study had two parts: an in vitro study and a clinical study. The study was in compliance with the Consolidation Standards of Reporting Trials and was registered on 16/9/2022 on Chinese Clinical Trial Registry (www.chictr.org.cn) (No. ChiCTR2200063800).

### Isolated tooth experiments

#### Specimen selection

To detect the impacts of two different scalers on a root surface, we chose subgingival calculus adjacent to the CEJ because the roughness of a dental crown is strongly affected by oral environmental factors. An in vitro study was conducted on 30 human tooth samples extracted from patients with severe periodontitis. The tooth samples were subjected to clinical and radiographic evaluations for severe periodontal disease—attachment loss of 5 mm or more, alveolar bone resorption greater than half of the root length, and loss of chewing function—by periodontists. Additionally, the tooth samples exhibited dental calculus in similar areas. The patient provided informed consent and signed consent forms for the use of the extracted teeth. The study received approval from the Research Ethics Committee of the School and Hospital of Stomatology, Fujian Medical University (No. 2020–51). The participants were randomly assigned to one of two experimental groups: the magnetostrictive scaler group (n = 15) (No. 1–15) or the piezoelectric scaler group (n = 15) (No. 16–30). The specific criteria for the required isolated teeth^2^ were as follows: the teeth must not have been previously scaled or root planed and must have intact surfaces, no caries, no fracture lines, and no obvious depressions. After extraction, the teeth were rinsed with running water, and the attached fibrous tissues were carefully removed and preserved in a thymol solution. The follow-up in vitro scaling was conducted by the same experienced periodontist (XQY). Another experienced clinician (XHW) evaluated the operation time and scaling effects. Both clinicians were specialists with more than ten years of experience using ultrasound equipment. The sample size for this experiment was calculated using the following formula:

$$ {\text{n}} = \frac{{\left( {Z_{\alpha } + Z_{\beta } } \right)^{2} * 2\sigma^{2} }}{{\delta^{2} }}, $$

where n is the sample size of each group (generally, if α is 0.05 and the Z value is two-sided, then Z0.05 = 1.96); β is the test efficiency (generally, if β is 0.9, then Z_β_ = 1.28); σ is the standard deviation, and δ is the difference between the two groups. The calculation is performed based on the Ra values after the scraping of the two devices in previous literature and the estimated difference. The calculations used the values reported by Busslinger and Yousefimanesh^9,18^, providing preliminary experimental results and data analysis. The sample size was fifteen.

#### Ultrasonic devices

**Figure 1:**
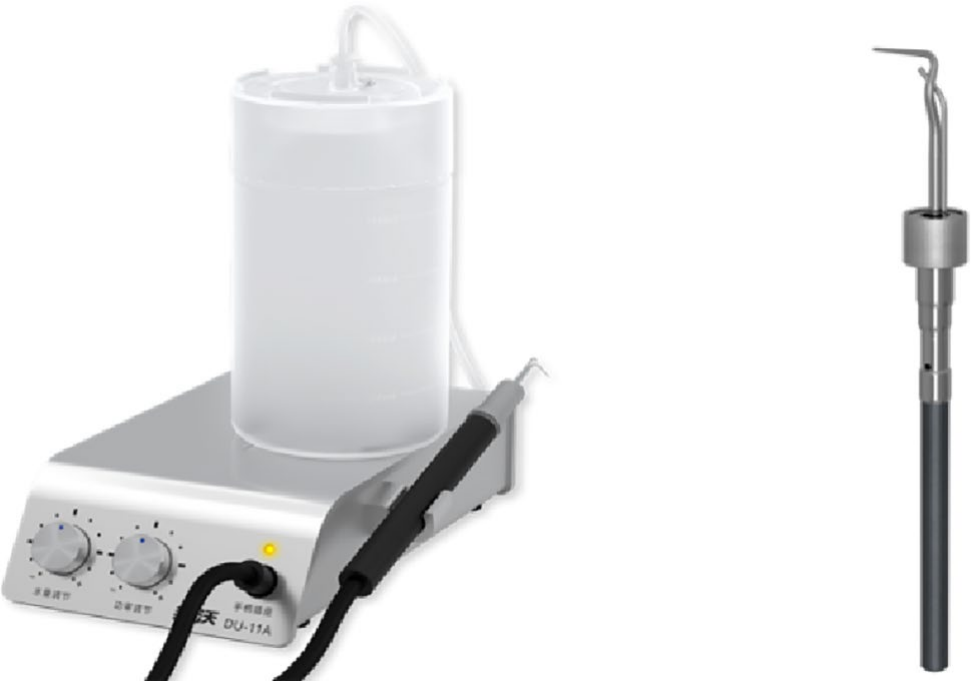
Magnetostrictive scaler (Bangvo). This image was approved for publication under the CC by Open Access Licence.

The roots were treated using a magnetostrictive ultrasonic scaler (DU-11A, Bangvo Technology Co., Ltd., China) with a perio (P23) tip (Fig. 1) and a piezoelectric ultrasonic scaler (Piezon Master 700, EMS, Switzerland) with a perio (P) tip. Both devices were stand-alone units with independent water supplies at room temperature during the treatment sessions. The power was initially set to 20% of the rated power, and it did not exceed 50%. The power was adjusted based on the difficulty of calculus removal. To prevent headpiece energy attenuation and tip wear, relatively new handpieces and ultrasonic tips with no more than 1 mm of wear were used to maintain the working efficiency.

#### Comparison of the scaling time between the two groups

**Figure 2:**
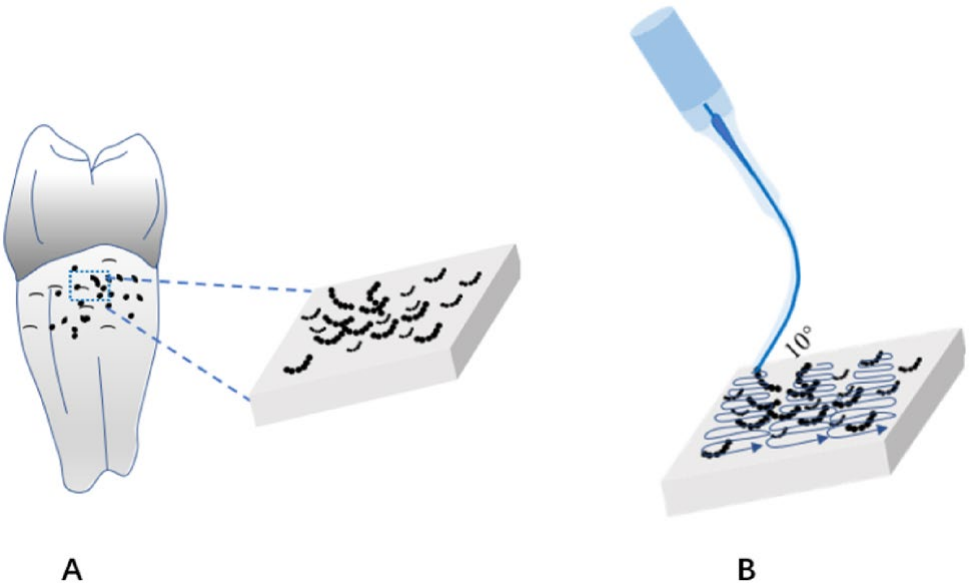
Schematic diagram of tooth preparation for the in vitro tooth experiment. (**A**) Area of 3 × 4 mm marked under the CEJ for subgingival scaling. (**B**) Working tip moving in the 'z' direction.

Herein, areas of calculus measuring approximately 4 × 3 mm on the root surface (n = 15, each group), located less than 1 mm from the cementoenamel junction (CEJ), were marked using diamond burs. The teeth were scaled similarly to their clinical application, with the working tip held at an angle of approximately 10° in the marked area and moved in a zigzag manner from the top to the bottom of the root (Fig. 2). The surface of interest was cleaned and smoothed by overlapping each movement until it met the visual and tactile criteria, as determined by a sharp explorer. The time taken to manipulate each surface to meet the above criteria was recorded. Throughout the study, the roots were held in a saline solution to prevent desiccation.

#### Tooth surface roughness measurement

The tooth surfaces of both groups (n = 15 each) were sliced into 4 × 3 mm sections using a diamond bur. The surface roughness was measured using a surface roughness meter (TIME TR-240, China) by determining the Ra value. The Ra value was measured thrice for each tooth section before and after scaling.

#### Tooth surface observation using SEM

The tooth pieces were fixed in a 4% paraformaldehyde solution for 48 h, gradually dehydrated, dried at the critical point, and sputtered with gold using a sputtering device. Three randomly selected samples per group were examined under a scanning electron microscope (ZEISS Sigma 300, Germany) at 200 × and 3000 × magnifications. The surface microstructure, smear layer, and calculus were detected with this microscope. In the magnetostrictive group, tooth pieces coded 5, 7, and 11 were selected. In the EMS group, tooth pieces coded 19, 20, and 29 were selected.

### Clinical experiments

#### Study design, sample size and patient selection

Eighty-five patients were enrolled in the study at a stomatological hospital affiliated with Fujian Medical University for periodontal treatment. The inclusion criteria were as follows^25^: (1) 18–65 years old; (2) at least 12 natural, vital teeth on the right and left sides of their mouth; (3) a calculus index (CI-S) score of 1–3 and a similar distribution of calculus on the right and left sides, with a diagnosis of gingivitis or mild periodontitis (PD ≤ 4 mm, CAL ≤ 1–2 mm); (4) no systemic diseases or coagulation dysfunction; (5) no dentin hypersensitivity, dental phobia, unfilled deep caries, or wedge-shaped defect; (6) not received periodontal treatment in the past six months; (7) no medical or psychological disease requiring the consumption of painkillers or antibiotics; (8) no acute infectious diseases of the teeth; (9) no smoking; (10) no pregnancy or menstruation in female patients; and (11) no drug abuse or alcoholism. This study was approved by the Research Ethics Committee of the School and Hospital of Stomatology, Fujian Medical University (No. 2020-51). Written informed consent was obtained from all participants before the study. The study adhered to the ethical standards of the 1964 Declaration of Helsinki. Trial registration: The sample size of the clinical studies was calculated according to the following formula:

$$ {\text{n}} = \left[ {\frac{{\left( {t_{{1 - {\raise0.7ex\hbox{$\alpha $} \!\mathord{\left/ {\vphantom {\alpha 2}}\right.\kern-0pt} \!\lower0.7ex\hbox{$2$}}}} + t_{1 - \beta } } \right)S}}{\delta }} \right]^{2} , $$

where n is the sample content required for a single group; S is the generally recognised standard deviation; δ is the difference with clinical significance; t_1-α/2_ is the first 1-α/2 quantile corresponding to the t distribution probability density curve (generally, α = 0.05); and t_1−β_ is generally 0.9 or 0.8. According to the previous literature, the estimated difference and standard deviation values of the two groups of VAS values were calculated. The corresponding values based on the preliminary experimental and data analysis results presented in published articles were used for the calculation^8,25,29^. The sample size was determined to be seventy-seven, accounting for a 10% loss to follow-up rate. Therefore, eighty-five subjects were included.

#### Ultrasonic devices and environmental controls

The magnetostrictive and piezoelectric ultrasonic devices were used with new working handles and tips that had no more than 1 mm of wear. The study maintained fixed environmental conditions to reduce their influence on patient perception. Effective doctor‒patient communication was ensured by having the same qualified dentist (XQY). Supragingival scaling was chosen as the experimental procedure to avoid the use of local anaesthesia and prevent pain caused by gingival injury, which could affect the accuracy of the results.

#### Treatment process

Prior to treatment, all subjects were given a detailed explanation of the study procedure and visual analogue scale (VAS) score and signed an informed consent form. The subjects first performed an air blow test for comfort. Two teeth in different quadrants of the subject were selected, and a puff of air was blown 2 cm from the neck of the tooth with an air gun on the chair. A single-centre blinded split-mouth comparison was performed, and the order of treatment was determined by a coin toss: the left or right half of each patient's mouth was randomly assigned to the magnetostrictive scaler (n = 85) or piezoelectric scaler group (n = 85), and the order of treatment for each modality was determined separately. The calculus was gently removed at a 10-degree angle to the tooth surface. After completing one half of the scaling, the operator would tell the patient the following statement: "One half of the scaling is completed, now we will start the treatment on the other half of the mouth." Then, the supragingival and 2-mm-deep subgingival calculus was completely removed in the left-to-right, bottom-to-top and labial–buccal sequence to lingual–palatal scaling. Both groups were operated on using standard techniques. The initial power was set to 20% of the nominal power. If the calculus was difficult to remove, the power was gradually increased but not to more than 50% of the nominal power. The water was regulated to a moderate degree. The same experienced periodontist (XQY) performed the randomised patient grouping and scaling, while the recorded treatment times and results were evaluated by another practitioner (XHW).

#### Questionnaire

**Figure 3:**
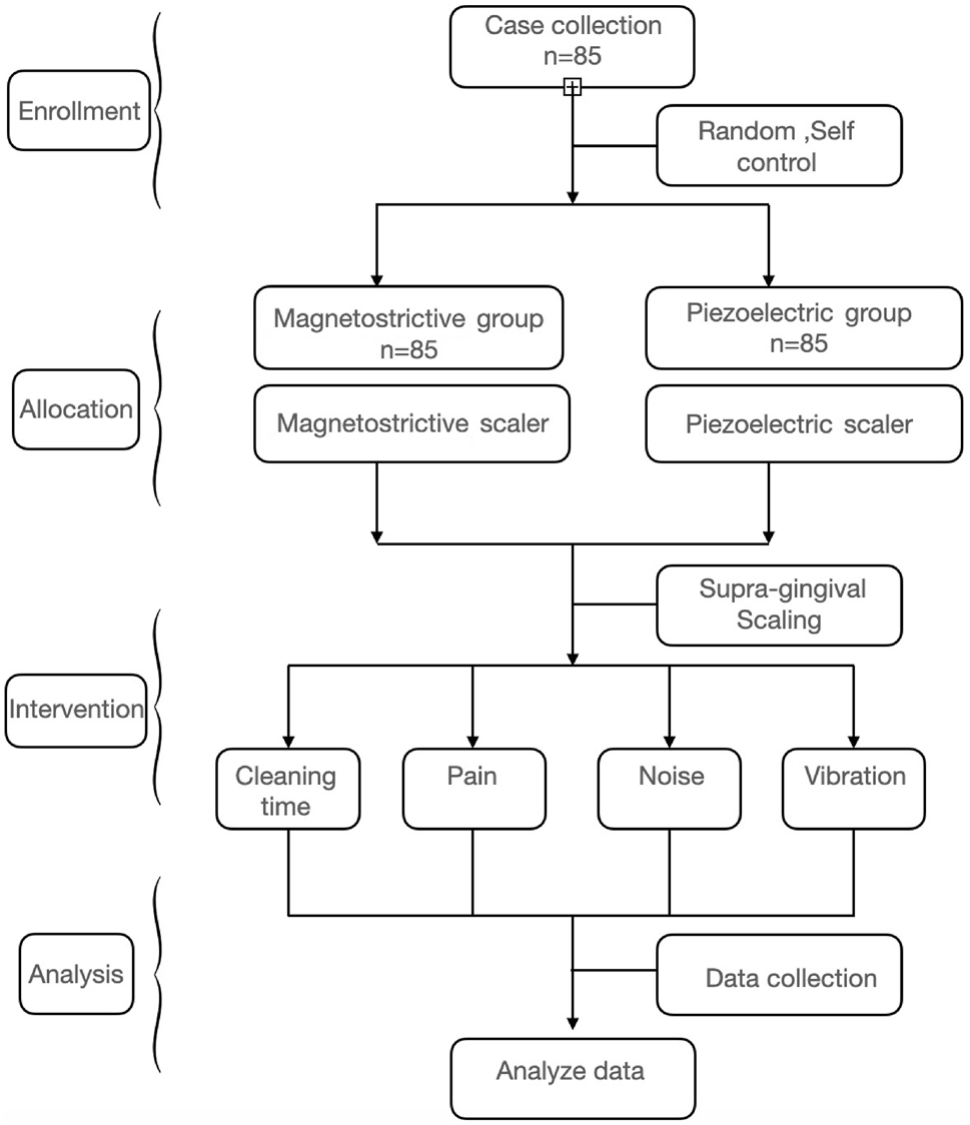
Flow chart of the clinical study.

After the subject completed the half-mouth treatment, we recorded the operation time and asked them to rate their level of tooth sensitivity, discomfort (defined as pain), vibration, and noise using a visual analogue scale (VAS). The visual analogue scale (VAS) used in this study was a horizontal continuous interval scale ranging from 0 to 100 mm, with 0 indicating 'no discomfort', 'no vibration', and 'no noise', and 100 indicating 'very severe tooth sensitivity', 'intolerable pain', 'intolerable vibration', and 'intolerable noise'. The scale represents progressively increasing degrees as the number of millimetres increases. After completing treatment on each side, another practitioner provided the subjects with a visual analogue scale (VAS) survey. The data were then stored properly. Figure 3 shows the flowchart of the design and execution of the clinical trials.

### Statistical methods

The data were analysed and processed using SPSS 24.0. The in vitro independent sample t test was conducted to evaluate the operation time and root surface roughness. The clinical experimental data were tested for normality using the Kolmogorov‒Smirnov (K–S) test. Since none of the data met the assumption of normality (P < 0.05), a nonparametric test was performed using the paired-samples sign test. The level of statistical significance was set to P < 0.05.

### Ethics approval

This study was approved by the Research Ethics Committee of the School and Hospital of Stomatology, Fujian Medical University (No. 2020–51). Written informed consent was obtained from all participants before the study. The study followed ethical standards as laid down in the 1964 Declaration of Helsinki. Trial registration: This clinical study followed the Consolidation Standards of Reporting Trials statement and was registered on 16/9/2022 on Chinese Clinical Trial Registry (www.chictr.org.cn) (No. ChiCTR2200063800).

## Results

### In vitro* study*

#### Operation time for isolated teeth

To remove calculus from the roots of similar areas, the mean operation times for piezoelectric or magnetostrictive scalers were 53.87 ± 7.6 s and 24 ± 3.42 s, respectively. The magnetostrictive scalers were more efficient than the piezoelectric scalers (P = 0.03).

#### Measurement of the tooth surface roughness

**Figure 4:**
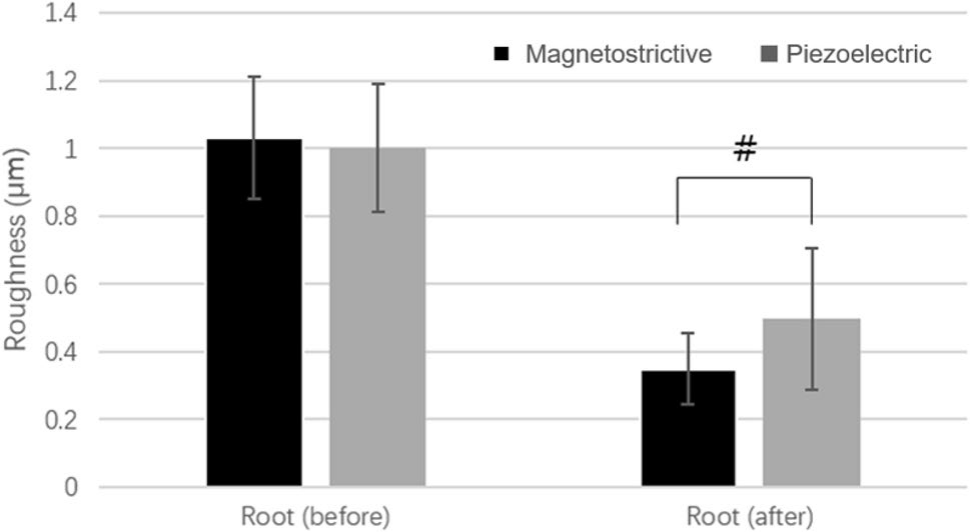
Mean roughnesses of the root surfaces before or after scaling with magnetostrictive or piezoelectric ultrasonic devices (^*#*^*P* < *0.05*).

The initial mean Ra values for the magnetostrictive scaler group and piezoelectric scaler group were 1.03 µm and 0.94 µm, respectively (P = 0.4). After scaling, the mean Ra values decreased to 0.349 µm for the magnetostrictive scaler group and 0.496 µm for the piezoelectric scaler group (P = 0.02, Fig. 4).

#### SEM observation of the tooth surface

**Figure 5:**
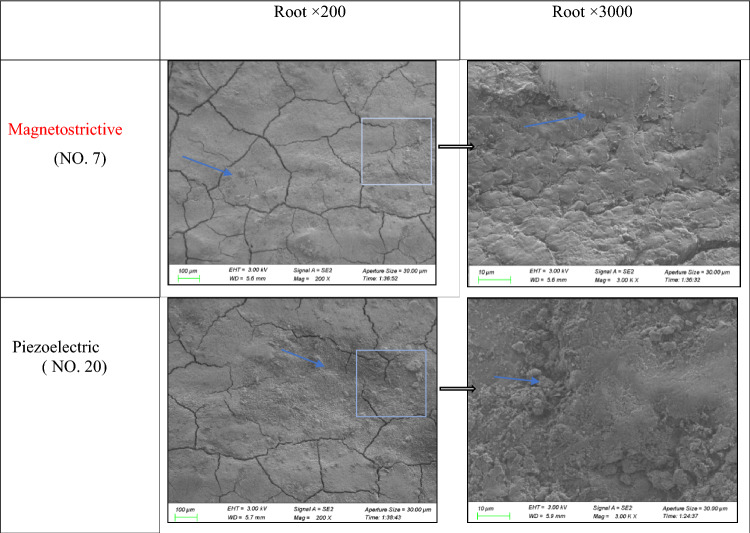
SEM images of root surfaces scaled with two different ultrasonic devices; some smear layers were left on the tooth surface (calculus and smear, layers 3000 ×  →  cementum exfoliation 3000 ×). There was more lost dental material and more smears retained in the piezoelectric group than in the magnetostrictive group.

SEM analysis revealed that both magnetostrictive and piezoelectric scalers were effective at removing calculus. However, some smear layers and calculus remained in both groups, particularly in the piezoelectric group. The surfaces scaled with magnetostrictive scalers were smoother than those scaled with piezoelectric scalers, reducing the loss of dental materials (Fig. 5).

### Clinical study

#### Sample characteristics and demographic data

Data from eighty-five subjects aged 18 to 63 years, including 35 males (41.2%) and 50 females (58.8%), were included. The patients were all from the Han population. A total of fifty-four patients (63.5%) were diagnosed with gingivitis, and thirty-one patients (36.5%) were diagnosed with mild periodontitis (Table 1).

**Table 1:** General information of the experimental cases.

Variables	Groups	n (%)
Classification of diseases	Mild periodontitis	54 (63.5%)
	Gingivitis	31 (36.5%)
Sex	Man	35 (41.2%)
	Woman	50 (58.8%)
Age bracket	18–30 years Old	37 (43.5%)
	30–50 years Old	39 (45.9%)
	50–63 years Old	9 (10.6%)

#### Comparison of operation times

The median operation time was 13 min for the Bangvo group and 15 min for the piezoelectric scaler group. The treatment time for the magnetostrictive scaler group was shorter than that for the piezoelectric scaler group (*P* < 0.05, Table 2).

**Table 2:** Comparison of operation times for both periodontal treatment devices.

Group	Median (min)	Z	*P*
Magnetostrictive	13	− 6.646	3.01 × 10^–11#^
Piezoelectric	15		

Since the data did not conform to normality, a paired-samples sign test was used for nonparametric tests.

^#^Statistically significant difference (*P* < 0.05).

#### Pain, noise, and vibration comparison

The median pain sensation was 40 for the magnetostrictive scaler group and 27 for the piezoelectric scaler group (*P* < 0.05). The median noise of the magnetostrictive scaler was 30, while the median noise of the piezoelectric scaler was 20 (*P* < 0.05). The median vibration of the magnetostrictive scaler group was 20, while that of the piezoelectric scaler group was 10 (*P* < 0.05). Therefore, the pain sensation, noise, and vibration sensation caused by the magnetostrictive scaler were all more intense than those caused by the piezoelectric scaler (*P* < 0.05, Table 3).

**Table 3:** Comparison of pain, noise, and vibration levels between periodontal treatment devices.

Variables	Groups	Median	Z	*P*
Pain	Magnetostrictive	40	− 7.651	2.0 × 10^–14#^
	Piezoelectric	27		
Noise	Magnetostrictive	30	− 5.769	7.97 × 10^–9#^
	piezoelectric	20		
Vibration	Magnetostrictive	20	− 4.854	1.0 × 10^–5#^
	Piezoelectric	10		

Since the data did not conform to normality, a paired-samples sign test was used for nonparametric tests.

^#^Statistically significant difference (*P* < 0.05).

#### Sex comparison

In the magnetostrictive scaler group, the median level of pain was 47.5 in women and 40 in men (*P* = 0.034). There were no differences in noise or vibration between the female and male groups (*P* > 0.05). For the piezoelectric scaler group, sex differences in pain, noise, and shock (*P* > 0.05) were not observed (Table 4).

**Table 4:** Effects of sex on pain sensation, noise, and vibration sensations.

Groups	Sense	Sex		Z	P
		Man	Woman		
Magnetostrictive	Pain	40	47.50	− 2.118	0.034
	Noise	30	30	− 0.637	0.524
	Vibration	20	20	− 0.558	0.577
Piezoelectric	Pain	25	28.50	− 0.059	0.953
	Noise	20	20	− 1.480	0.139

Since the data did not conform to normality, a paired-samples sign test was used for nonparametric tests.

#### Comparison between different diagnoses

In the magnetostrictive scaler group, the median pain was 45 for the mild periodontitis group and 40 for the gingivitis group (*P* = 0.045). There were no differences in noise or vibration among the patients with the different diagnoses (*P* > 0.05). In the piezoelectric scaler group, pain, noise, and vibration variations were not significantly different among the patients with different diagnoses (*P* > 0.05, Table 5).

**Table 5:** Effects of different diagnoses on pain sensation, noise, and vibration sensation.

Group	Sense	Diagnosis		Z	*P*
		Mild periodontitis	Gingivitis		
Magnetostrictive	Pain	45	40	− 2.003	0.045^#^
	Noise	30	25	− 0.921	0.357
	Vibration	20	20	− 1.353	0.176
Piezoelectric	Pain	30	20	− 1.725	0.085
	Noise	20	20	− 1.375	0.169
	Vibration	17.50	10	− 0.931	0.352

Since the data did not conform to normality, paired-samples sign tests were used for nonparametric tests.

^#^Statistically significant difference (*P* < 0.05).

## Discussion

An ultrasonic scaler is composed of an ultrasonic generator and a transducer. The generator produces an electric or magnetostrictive field, and the transducer converts high-frequency electric or electromagnetic energy into ultrasonic vibrations. However, the mechanical friction of the scaler tips can cause scratches or minor damage to the tooth surface^29^. Furthermore, ultrasonic scaling produces noise and vibration, which may cause temporary discomfort or pain to patients. Although periodontal debridement has several shortcomings, it is still a widely used and efficient method. In this study, the null hypothesis in which magnetostrictive and piezoelectric ultrasonic scalers result in the same root surface morphologies and comfort levels during supragingival debridement is rejected.

The results of the in vitro experiment indicate that the magnetostrictive scaler has a greater scaling efficiency than the piezoelectric scaler, especially in removing calculus deposits from the root surface. This finding is consistent with clinical studies and is supported by the results of Yousefimanesh^18^. During the treatment, the tips of the ultrasonic scaler were maintained at a 10-degree angle from the tooth surface. However, this placement did not guarantee similar working performance due to differences in working principles. In previous studies, it was confirmed that the higher the output power of the scaler is, the more effective it is^30^. Therefore, it is possible that the output power of the magnetostrictive scaler is greater than that of the piezoelectric scaler. There are differences in the shapes of the different ultrasonic scaler tips. The piezoelectric scaler working tip is flat and in tangential contact with the root surface, while the magnetostrictive scaler working tip is conical, resulting in a relatively large contact area with the tooth surface. However, Busslinger et al.^9^ demonstrated that the scaling efficiency of piezoelectric ultrasonic scalers is significantly greater than that of magnetostrictive ultrasonic scalers. This difference in results may be attributed to variations in the brands, working tips, and lateral forces of the periodontal instruments used in our experiments^29–31^.

A magnetostrictive scaler produces a smoother tooth surface than a piezoelectric scaler. Notably, this experiment is randomised, and therefore, the roughness observed can be attributed to surface deposits or scratches formed on the tooth surface or to the removal of cementum during ultrasonic scaling. This difference in the working tip may have contributed to the observed results. The magnetostrictive scaler (Bangvo) working tip is made of titanium alloy, which is less prone to wear and better for protecting the root surface than that of the piezoelectric scaler. In contrast, the piezoelectric scaler working tip is made of stainless steel, which is harder to wear and more likely to damage the root surface than titanium alloy. These results are consistent with those of Busslinger et al.^9^ and Mahiroglu et al.^32^, who reported that a piezoelectric scaler produces a rougher root surface than a magnetostrictive group. In contrast, Yousefimanesh et al.^18^ reported that a piezoelectric scaler produces a smoother tooth surface than a magnetostrictive scaler when using the same 200-g lateral forces. Mittal et al.^19^ reported that while the root surface scaled with a piezoelectric scaler is less rough than that scaled with a magnetostrictive scaler, it loses more material and has more noticeable scratches. Singh et al.^20^ and Brine et al.^33^ reported that magnetostrictive and piezoelectric ultrasonic scalers produce similar surface roughnesses, differing from the studies mentioned above. The differences in the results likely occur due to the use of different ultrasound devices. Additionally, another scholar^17^ reported that tooth surface defects caused by dental ultrasonic scalers are not dependent on whether the scaler is magnetostrictive or piezoelectric. There is ongoing debate regarding whether a rough or smooth surface is preferable. However, most studies suggest that smooth surfaces are more conducive to the proliferation of gingival and periodontal ligament cells and the reduction in bacterial deposition than rough surfaces. Although a few studies have reported that fibroblasts favour a rough root surface^22^, the condition of the root surface may be a contributing factor, given the conflicting data in previous reports. Therefore, electron microscopic observation is necessary to determine the main cause of the variable roughness.

The results of the SEM analysis indicate that the magnetostrictive scaler (No. 7) produces smoother tooth surfaces and causes less tooth material loss than the EMS scalers (No. 20). This result suggests that the difference in roughness may be related to root surface wear. Chiesa et al. reported that changes in the surfaces of artificial or natural teeth depend on the surface being treated, while the type of powder and granulometry used do not affect the observed lesions^34^. In this study, the treated surfaces are exclusively root surfaces. Therefore, the observed differences may be attributed to variations in the working methods and manufacturing materials of the two scaler tips. Additionally, both groups exhibit residual smear layers and calculus. It is suggested that ultrasonic scalers alone may not be sufficient to completely remove smear layers from the root surface. Additional measures, such as manual scalers, lasers, and endoscopic periodontal treatment, may be necessary to achieve complete scaling.

In a clinical study, patients have reported that the pain, noise and vibration caused by magnetostrictive scalers are greater than those caused by piezoelectric scalers. The difference in the data between the two devices can be attributed to the different movements of their working tips. The piezoelectric scaler has a linear movement, with the working tip moving back and forth in a longitudinal plane and being active laterally. The magnetostrictive scaler exhibits more elliptical movement than the piezoelectric scaler, with multiple planes moving horizontally and longitudinally. The scaler tip is activated on all surfaces, resulting in a stronger tapping and hammering sensation. Currently, there is no uniform method for quantifying the output power of a machine, making it difficult to compare two machines of the same power. To reduce discomfort and loss of hard tissue, low power is recommended^31^. The piezoelectric scaler has a negative feedback function, which automatically adjusts the output power based on the resistance encountered during the movement of the working tip. Furthermore, different working tip designs result in unique changes on the tooth surface^32^. Additionally, the magnetostrictive instrument has a stronger working tip than the piezoelectric instrument, which may cause additional discomfort. Therefore, it has been suggested^35^ that using slender working tips may reduce pain and tooth material loss. Müller et al.^36^ also reported that piezoelectric ultrasonic scalers cause less discomfort in terms of pain, noise, and vibration than magnetostrictive ultrasonic scalers. Another study^14^ showed no significant differences between the two types of devices when room temperature water is used. However, when warm water is used, the piezoelectric device provides a better experience than the magnetostrictive device. Kocher et al.^1^ reported no difference in patient experience between the two devices. However, a study by Ikeda et al.^26^ suggested that patients prefer magnetostrictive devices. The piezoelectric scaler produces low pain intensity in this study, making it suitable for sensitive teeth. These experimental results indicate that the level of patient discomfort may not be proportional to tooth surface roughness or damage but rather to the different working principles of the devices.

Women experience more discomfort than men with magnetostrictive scalers, and patients with mild periodontitis experience more pain than those with gingivitis. Karadottir et al.^37^ also reported that women have greater fear, anxiety and pain responses than men in terms of several measures. However, there are no differences between the sexes in the experience of noise and vibration. Nevertheless, there are no sex or diagnostic differences resulting from the use of piezoelectric scalers. This study shows that patients with mild periodontitis have shrunken gums with partially exposed root surfaces, which may be due to the weakness of the root cementum compared to that of the enamel. This weakness makes the root cementum highly sensitive to external stimuli, resulting in increased pain during extensive multipoint contact with the magnetostrictive scaler.

However, this study has certain limitations. Muhney et al.^25^ recommended that samples with similar pain thresholds or acoustic sensitivities should be included in the inclusion criteria, which is not considered in this study. Due to the different principles of the instruments, it is difficult to ensure consistent power output and lateral force during scaling in the in vitro study. In the clinical study, only supragingival scaling is evaluated. Future studies should consider performing an in-depth analysis of scaling and using digital devices to indicate pain intensity and ensure consistent instrument force during treatment. Notably, in vitro experiments cannot fully simulate clinical trials. Further research is required to investigate the growth of periodontal cells on scaled root surfaces, as the differences between the two ultrasonic scalers have only been evaluated by the roughness and surface changes in the teeth.

## Conclusions

According to this study, the Bangvo scaler is more effective and causes less damage to tooth surfaces during supragingival scaling than the EMS scaler in vitro. However, the EMS scaler causes less discomfort than the Bangvo scaler in clinical trials.

In summary, while piezoelectric scalers result in reduced levels of pain, noise, and vibration for patients, magnetostrictive scalers are more efficient and minimally invasive. The magnetostrictive scaler is more advantageous than the piezoelectric scaler for removing calculus with minor tooth damage and increasing the operator work efficiency. However, piezoelectric scalers are preferred for reducing patient discomfort. When selecting ultrasound treatment instruments in clinical practice, it is important to consider the actual situation.

## Data Availability

The complete data used in this study are available from the corresponding author upon request.
